# Cell-Free DNA Bisulfite Sequencing Reveals Epithelial–Mesenchymal Transition Signatures for Breast Cancer

**DOI:** 10.3390/ijms26178723

**Published:** 2025-09-07

**Authors:** Minsun Stacey Jeon, Zehuan Ding, Casey Pei, Jing Li, Linglin Xie, Edward Sauter, Ke Kurt Zhang

**Affiliations:** 1 Center for Epigenetics and Disease Prevention, Institute of Biosciences & Technology, Texas A&M Health Science Center, Houston, TX 77030, USA; minsunjeon@tamu.edu (M.S.J.); zehuan.d@tamu.edu (Z.D.); jing.li@tamu.edu (J.L.); 2 Rigor and Reproducibility Core, Institute of Biosciences & Technology, Texas A&M Health Science Center, Houston, TX 77030, USA; caseypei@tamu.edu; 3 Department of Nutrition, Texas A&M University, College Station, TX 77843, USA; linglin.xie@ag.tamu.edu; 4 National Cancer Institute, Division of Cancer Prevention, Rockville, MD 20850, USA

**Keywords:** breast cancer, DNA methylation, cell-free DNA, nipple aspirate fluid, whole-genome bisulfite sequencing, differentially methylated regions, epithelial–mesenchymal transition

## Abstract

Cell-free DNA (cfDNA), shed by malignant tumor cells into extracellular fluid, provides valuable epigenetic information indicative of cancer status. Nipple aspirate fluid (NAF), a noninvasive liquid biopsy from at-risk women, contains nucleic acid and protein biomarkers from adjacent cancer cells, showing promise for breast cancer (BrC) detection. However, despite its potential, the application of cfDNA in NAF for BrC screening is still underexplored. Here, we report a proof-of-concept study for using cfDNA bisulfite sequencing (cfBS) to assess tumor DNA methylation signatures from NAF samples. For four healthy individuals and three BrC patients, cfBS achieved greater than 20× sequencing depth with an average coverage of 26.5× on the genome. A total of 7471 differentially methylated regions were identified, with significant hypermethylation in BrC samples compared to healthy controls. Gene set enrichment analysis indicated that the differentially methylated genes (DMGs) were significantly associated with epithelial–mesenchymal transition (EMT). By developing a novel EMT scoring metric, we found that BrC samples had more of a mesenchymal phenotype than samples from healthy individuals. *CDH1*, *WNT2*, and *TRIM29* were hypermethylated near the promoter region, while *COL5A2* was hypermethylated in the coding region. The DNA methylation and EMT changes were validated through The Cancer Genome Atlas Breast Invasive Carcinoma study, which confirmed that DMGs were associated with gene expression change and that our methylation-based EMT score reliably distinguished tumors from healthy controls. Our findings support the utilization of the NAF cfDNA cfBS methylation profile for noninvasive BrC screening and pave the way for enhanced early detection of this disease.

## 1. Introduction

While mammography is the primary screening method for breast cancer (BrC), suspicious mammographic findings are often found on biopsy to be falsely positive [1,2,3,4]. The invasive procedures to exclude cancer are costly and can cause sequelae, some serious. Cell-free DNA (cfDNA) has been studied for its potential as a liquid biopsy for cancer screening and detection [5,6]. In particular, aberrant hypermethylation in tumor DNA has been found in the promoter regions of tumor suppressor genes, leading to their transcriptional silencing [7]. Detecting aberrant DNA methylation in plasma cfDNA has been explored in detecting early-stage BrC, particularly in individuals with dense breasts [8,9,10,11]. Genome-wide profiling demonstrated that 70% of tumor suppressor gene promoters, located within CpG islands, are hypermethylated [12,13]. Specifically, aberrant methylation was identified in the promoters of BrC-associated genes, including *APC*, *BRCA1*, and *RASSF1A*. [14,15,16]. Nevertheless, plasma cfDNA has shown fewer encouraging outcomes in BrC detection compared to other cancers [17], probably due to the low yield of cfDNA extracted from plasma [18].

Compared to plasma-based approaches, nipple aspirate fluid (NAF) provides a more localized and potentially enriched source of BrC-specific biomarkers [19,20]. Nipple aspiration is a safe, non-invasive procedure for collecting breast epithelial cells and extracellular fluid produced by the breast epithelium, which is predominantly where BrCs originate. NAF is a valuable source for liquid biopsy, particularly for the analysis of large biomolecules such as secreted proteins and DNA [21,22], though its relatively low cellularity makes it less ideal for cytologic evaluation [23,24]. cfDNA can be extracted from NAF, containing extracellular DNA fragments originating directly from the breast ductal system and the diseased tissue of origin. Therefore, cfDNA methylation in NAF has the potential to serve as a favorable and sensitive approach for BrC detection and screening.

Because NAF cfDNA is usually available in low quantities, a highly sensitive method is needed to detect tumor-derived signals. Whole genome bisulfite sequencing (WGBS) represents a state-of-the-art technology for DNA methylation analysis [25], providing genome-wide profiling at single-base resolution. Based on WGBS, we developed a low-input cfDNA bisulfite sequencing (cfBS) protocol optimized for NAF samples. Here, we present a proof-of-concept study to determine whether DNA methylation signatures derived from NAF cfBS could support accurate and reliable non-invasive BrC screening. Our approach successfully uncovered cfDNA methylation signatures in NAF from BrC patients, underscoring the promise of cfDNA methylome profiling as a novel tool for early detection.

## 2. Results

### 2.1. Characteristics of NAF cfBS

NAF samples were collected from seven individuals, three with BrC and four without. Sample volumes ranged from 2 to 20 µL. After cfDNA extraction and bisulfite conversion, a DNA library was constructed with roughly 20 ng of DNA per sample. The cfDNA libraries exhibited an average fragment size of 357 bp, and no significant adapter dimer peaks were observed. Each sample generated an average of 136 million reads per library. These reads were aligned to the hg38 reference genome, resulting in a mapping rate of over 97% for each sample (Table 1). The sequencing depth for each of the seven samples ranged between 20× and 35×, covering from 38.4% to 58.8% of the genome. These high mapping rates and high genome coverage demonstrated that high-quality cfBS data can be reliably obtained from a relatively small volume of NAF samples, affirming the feasibility of our approach for this proof-of-concept study.

We assessed the percentage of cfDNA sequenced reads mapped to different genomic regions in each sample to understand their genomic distribution. This analysis demonstrated a consistent pattern across the samples, with an average of 66.5% of the reads located within the gene body regions, as shown in Supplemental File S1: Figure S1a. A similar trend was observed for the distribution of CpG sites, where 72.1% were found in the gene body (Supplemental File S1: Figure S1b). The distribution pattern of cfDNA fragments suggests that they are not randomly distributed but are instead preferentially derived from gene regions. This pattern supports the reliability of NAF cfDNA as a source for quantifying DNA methylations that regulate gene expression.

### 2.2. Differentially Methylated Regions Are Enriched in Promoter Regions

**Figure 1:**
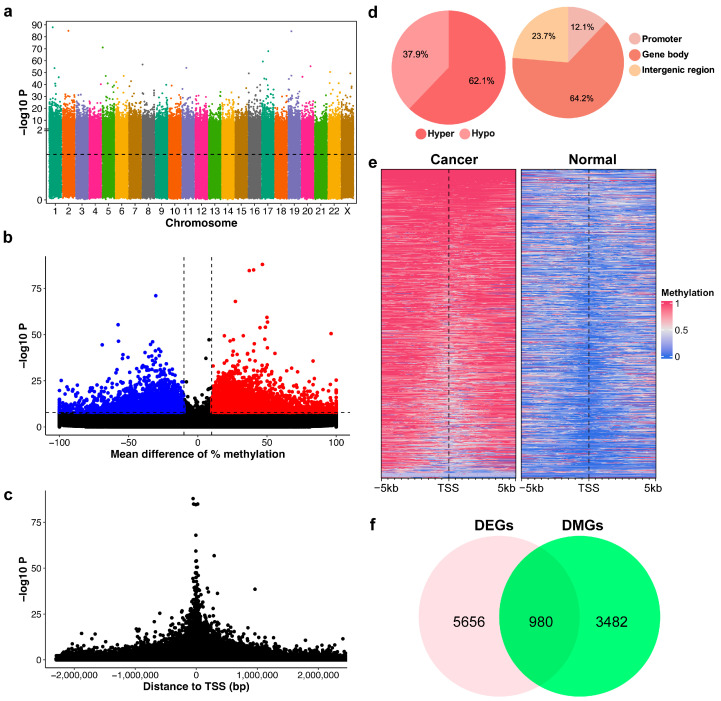
Characterization of DNA methylation patterns in breast cancer (BrC). (**a**) Manhattan plot showing the significance of differential methylation for CpG regions. The black dotted line indicates the threshold for significance (*p*-value < 0.05). (**b**) Volcano plot of the mean difference between normal and cancer samples versus the *p*-value. Dashed lines indicate cut-off for differentially methylated regions (DMRs)—abs mean difference ≥ 10%, *p*-value < 10^−8^, blue = hypomethylation, red = hypermethylation. (**c**) The relative distance of CpG regions to the transcription start site (TSS) versus the *p*-value. (**d**) Frequency of hypo- and hyper-DMRs; distribution of DMRs in the genome. (**e**) Methylation profile heatmap around the TSS of top significant genes. (**f**) Venn diagram showing the overlap between differentially expressed genes (DEGs) in BrC with differentially methylated genes (DMGs).

In order to identify differentially methylated regions (DMRs), we developed an unsupervised learning approach based on the mean shift algorithm to uncover CpG islands (methylated regions). Our algorithm identified DNA methylation regions with an average size of 246.61 bp and 10.14 CpG sites. This region size aligns closely with the standard definition of a CpG island (length ≥ 200 bp) as described by [26]. As a result, we identified a total of 3,493,780 methylation regions (Figure 1a).

Using Fisher’s exact test, we identified 7471 DMRs that exhibited greater than 10% methylation differences between cancer and normal samples and *p*-values less than 10^−8^ (Figure 1b). A volcano plot illustrates these methylation differences against their corresponding *p*-values, indicating a trend where statistical significance enhances with an increase in methylation difference (Figure 1b). The distribution of *p*-values relative to the transcription start site (TSS) exhibited a symmetric pattern, with increasing significance when closer to the TSS, as demonstrated in Figure 1c. Our results also highlighted a predominance of hypermethylated DMRs over hypomethylated ones, with 62.1% hypermethylated DMRs and 37.9% hypomethylated DMRs (Figure 1d). A substantial proportion of DMRs (64.2%) were located within gene regions, which typically comprise only 1–2% of the human genome. This indicates a significant enrichment of DNA methylation changes associated with genes, particularly in the promoter region (12.1%). In addition, distinct methylation patterns around the TSS were observed by contrasting normal and cancer samples, as shown in Figure 1e.

### 2.3. Differentially Methylated Genes Are Associated with Differentially Expressed Genes in BrC

Next, we investigated whether these identified DMRs contribute to gene expression alterations in BrC patients. We annotated differentially methylated genes (DMGs) by associating DMRs with their corresponding genes. This led to the identification of 5700 DMRs connected to the promoter and gene body regions of 4462 distinct genes. The top 50 DMRs and their associated DMGs are listed in Supplemental File S2: Table S1 To pinpoint specific genes implicated in BrC, we compared these DMGs with differentially expressed genes (DEGs) assessed from The Cancer Genome Atlas Breast Invasive Carcinoma (TCGA-BRCA) dataset. There were 6636 DEGs identified by comparing primary tumor tissues and normal solid tissues in the TCGA-BRCA dataset. Notably, there was an overlap of 980 genes between the DEGs and DMGs, as illustrated in Figure 1f. This overlap was found to be statistically significant (*p* < 1 × 10^−12^), as the observed number of overlapping genes exceeded those expected from random overlap. A total of 59.5% of these overlapping genes had an inverse correlation between DNA methylation and gene expression patterns. This result suggests that changes in DNA methylation could be a key molecular mechanism influencing gene expression alterations during BrC development.

### 2.4. Epithelial–Mesenchymal Transition Is Activated in BrC cfBS Data

**Figure 2:**
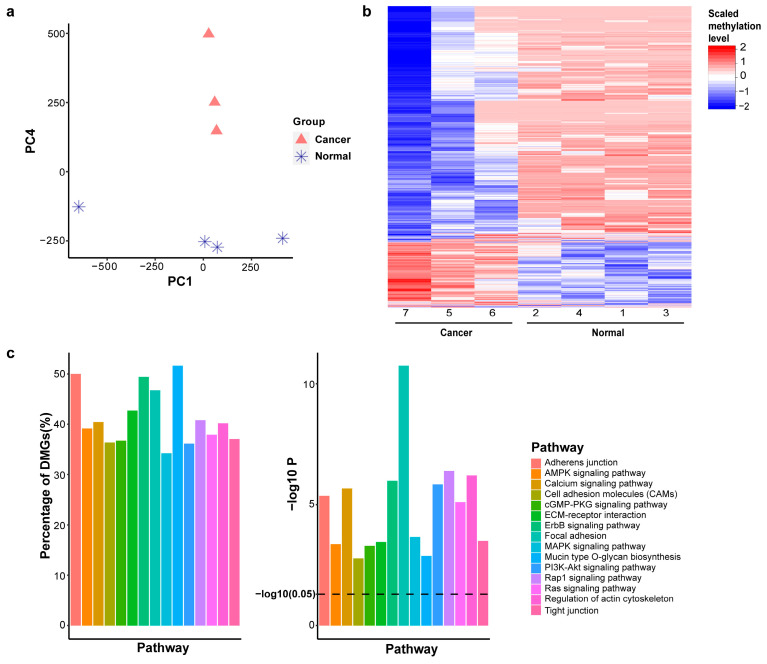
Principal component analysis reveals epithelial–mesenchymal transition (EMT). (**a**). Hierarchical clustering of DNA methylation profiles. (**b**). Heatmap of dominant genes in PC4. (**c**). Top 15 cancer-related pathways (q-value < 0.01), showing the percentage and *p*-value of each pathway.

To enhance our understanding of the relationships among the samples, principal component analysis (PCA) was conducted to display the positions of individual samples within a reduced dimensionality space. Notably, the first six principal components (PCs) represented almost equal variance (ranging from 274.3 to 317.0) and together accounted for more than 99.99% of the total variance in the data. By assessing each PC with respect to the two major groups, PC4 emerged as significant and informative, effectively differentiating between cancer and normal samples (Figure 2a). We selected the top 2.5% of CpG regions (PC4 weights > 0.0027). The threshold of 2.5% is based on the cutoff of a two-tailed statistical test at an alpha level of 5%. This selection resulted in 11,281 CpG regions. A heatmap of their methylation levels demonstrated that these genes are differentially methylated between the two groups, normal and cancer (Figure 2b). Gene set analysis of these PC4-associated genes highlighted the epithelial–mesenchymal transition (EMT)-related pathways, such as adherens junction, cell adhesion molecules, focal adhesion, PI3K-Akt signaling pathway, and Ras signaling pathway, to be significantly enriched (adjusted *p*-value < 0.01), suggesting EMT may play an important role in BrC development (Figure 2c).

**Figure 3:**
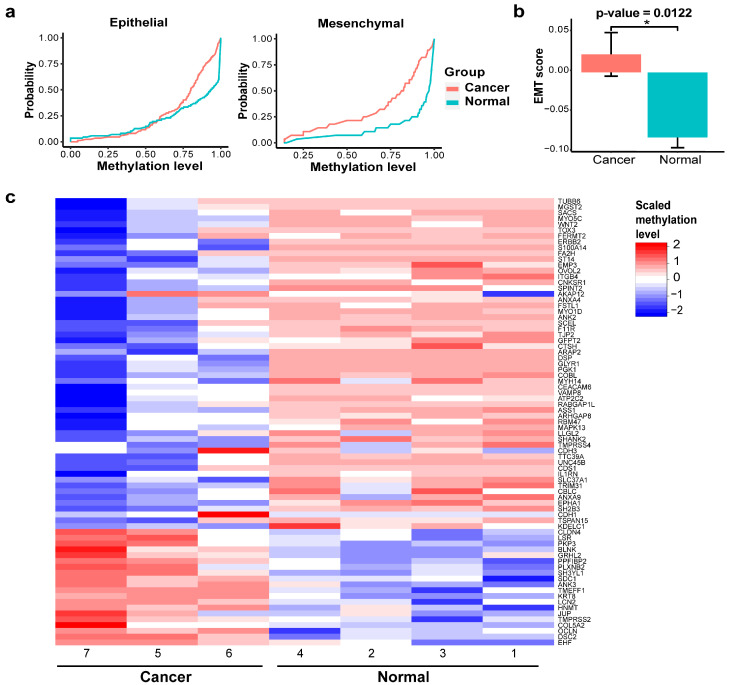
EMT status of BrC. (**a**) Epithelial (left) and mesenchymal (right) area under the curve (AUC). (**b**) EMT score between cancer and normal groups (* *p*-value < 0.05). (**c**) Heatmap of EMT gene signatures.

In order to study the EMT status of each sample, we developed EMT scoring metrics, which allowed us to quantify the extent of EMT-associated methylation changes. The methylation regions found in PC4 were linked to an EMT signature of 77 genes. The accumulated probability of methylation levels for either epithelial genes or mesenchymal genes was plotted in Figure 3a. The area under the curve (AUC) represents the distribution of these methylation levels. While the AUC for epithelial genes was similar between the two groups, the AUC for mesenchymal genes was higher in the cancer group compared to the normal group, indicating hypomethylation of mesenchymal genes in BrC. This pattern suggests that EMT is active in BrC, with cancer cells likely shifting toward a mesenchymal state (Figure 3a). EMT scores for BrC samples were significantly higher (more mesenchymal) when compared to the normal samples (Figure 3b; *p*-value = 0.0122). The methylation levels of these 77 EMT genes were demonstrated in the heatmap (Figure 3c). In BrC patients, 23.5% of genes were hypermethylated, while 76.5% were hypomethylated.

### 2.5. Validation of EMT Scoring Metric in TCGA Data

**Figure 4:**
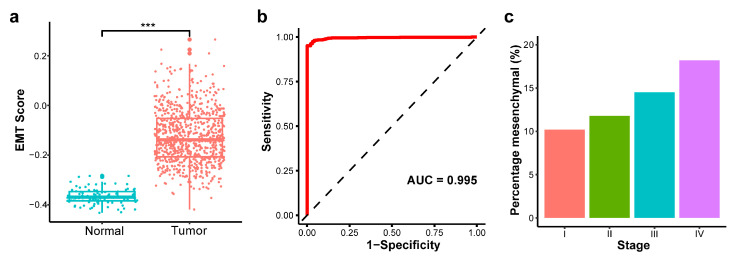
Validation of EMT scoring method in The Cancer Genome Atlas Breast Invasive Carcinoma (TCGA-BRCA) dataset. (**a**) EMT score between primary tumor and normal solid tissue (*** *p*-value < 0.001). (**b**) Receiver operating characteristic (ROC) curve for EMT score discriminating tumor versus normal samples. (**c**) Percentage of mesenchymal samples by tumor stage.

Applying the EMT scoring metric for the normalized methylation levels in TCGA, we found that the EMT scores for tumor samples were significantly higher than those of normal samples (Figure 4a; *p*-value < 0.001), indicating a predominantly mesenchymal state in cancer cells. We further evaluated the discriminative power of the EMT score by constructing a receiver operating characteristic (ROC) curve. The EMT score yielded an AUC of 0.995, demonstrating an almost perfect ability to distinguish tumor samples from normal controls (Figure 4b). The TCGA primary tumor samples were classified as stages I through IV, where lower stages signify that the cancer is more localized and has not spread extensively, reflecting a potentially earlier phase of the disease. We calculated the percentage of mesenchymal samples (EMT score > 0) for each tumor stage and observed an increase in the proportion of mesenchymal samples as the tumor stage advanced (Figure 4c).

### 2.6. EMT Gene Expression and Methylation Correlations in TCGA Data

**Figure 5:**
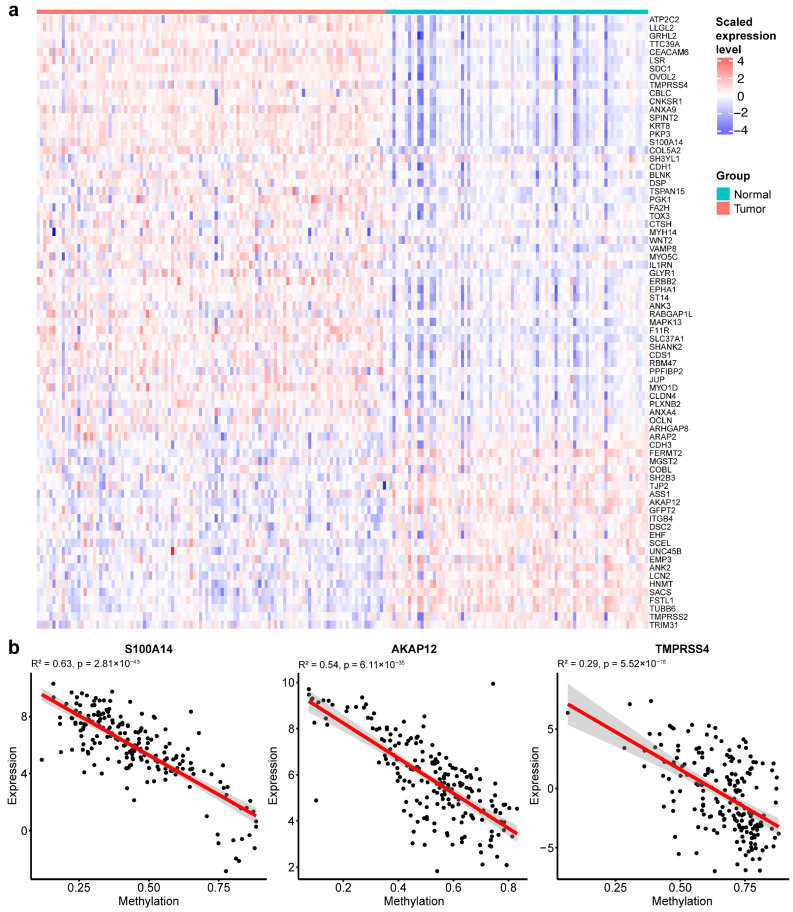
EMT gene expression and methylation correlations in TCGA-BRCA. (**a**) Expression heatmap of EMT gene signatures in TCGA-BRCA samples. (**b**) Representative scatter plots showing correlations between DNA methylation levels and gene expression levels for EMT genes.

To evaluate the expression of the 77 EMT genes, we plotted the heatmap in the matched subset of TCGA tumor and normal samples (Figure 5a). Among the 77 genes, there were 64 genes (83.1%) found differentially expressed in the TCGA study, and 68.7% of genes were upregulated while 31.3% of genes were downregulated in the cancer group.

We further assessed the relationship between gene expression and DNA methylation. Several EMT genes, including *S100A14*, *AKAP12*, and *TMPRSS4*, displayed inverse correlations between methylation and expression (Figure 5b). These findings demonstrate widespread EMT-associated transcriptional changes in BrC tumors and suggest that DNA methylation may serve as a potential regulatory mechanism of EMT.

### 2.7. NAF cfDNA Methylation Reflects Tumor-Specific EMT Gene Alterations

To validate our cfDNA findings, we examined EMT-associated genes that showed matched methylation and expression changes in TCGA and assessed whether similar methylation changes were observed in NAF cfDNA. *RABGAP1L* displayed predominant hypomethylation and was upregulated in primary tumor samples, with cfDNA methylation analysis confirming hypomethylation within intron 17 (748 bp). In contrast, *AKAP12* exhibited hypermethylation and was downregulated in breast tumors, with cfDNA analysis confirming hypermethylation within intron 2 (329 bp) in NAF BrC samples. *PGK1* was hypomethylated and upregulated in BrC samples, with cfDNA analysis also revealing hypomethylation in intron 1 (716 bp). Additionally, *S100A14* and *TMPRSS4* were both hypomethylated and upregulated in breast tumors. cfDNA analysis revealed hypomethylation in the promoter region of *S100A14* (528 bp) and in intron 1 of *TMPRSS4* (736 bp).

### 2.8. Differential Methylation of EMT Genes

**Figure 6:**
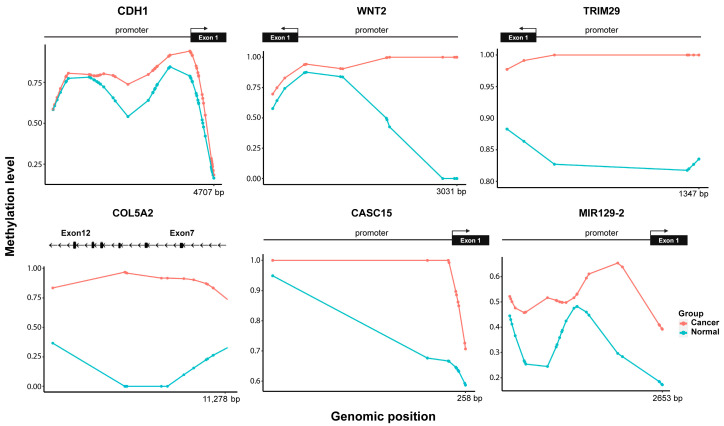
Methylation status of CpG sites of genes associated with BrC and EMT. Hypermethylation observed for the cancer group (red) in the promoter and gene body regions.

We confirmed the DNA methylation variations in select genes that were associated with BrC and EMT at the CpG level in NAF samples. Specifically, *CDH1*, *WNT2*, and *TRIM29* were hypermethylated near the promoter region, while *COL5A2* was hypermethylated in the coding region (Figure 6), with methylation levels of 25%, 100%, 17.5%, and 100%, respectively. Beyond protein-coding genes, our methylation analysis also highlighted CpG sites with the highest *p*-values mapped to noncoding regions, particularly in the long noncoding RNA (lncRNA), CASC15 and miR-129-2. Interestingly, hypermethylation was observed upstream of the TSS of both genes (within 5000 bp), with methylation levels of 30% for both (Figure 6), suggesting potential regulatory implications.

## 3. Discussion

Early and accurate detection of BrC is essential to improve patient survival. Nevertheless, current screening strategies like mammography have known limitations, including reduced sensitivity and a higher risk of overdiagnosis. This study provides proof-of-concept evidence that NAF can serve as a noninvasive liquid biopsy for BrC detection. NAF offers a promising source of cfDNA for comprehensive genome-wide DNA methylation analysis. This is not the first study to evaluate DNA methylation in NAF, with prior studies by ourselves [27,28] and others [29] using methylation-specific PCR (MSP). In this report, we discuss our findings using cfBS, generally considered the “gold standard” method of DNA methylation assessment [25], for it offers a comprehensive, quantitative analysis of methylation across a region of DNA, whereas MSP is a qualitative method which focuses on identifying methylation at specific CpG sites. Using cfBS, we demonstrate the feasibility of obtaining high-quality, informative methylation data from minimal sample volumes (2–20 µL), the typical amounts of NAF obtained in clinical settings. In this study, we successfully extracted 0.6–3.4 ng of cfDNA from less than 20 µL of NAF. Through cfBS, we generated high-quality libraries, confirming the technical feasibility and reliability of using NAF-derived cfDNA for methylation-based analysis, supporting its potential as a minimally invasive platform for BrC screening and molecular characterization.

For early cancer diagnosis, high-efficiency library construction and sensitive cfDNA detection are necessary yet challenging due to the limited yield and highly fragmented nature of cfDNA [30]. While DNA methylation biomarkers derived from cfDNA have shown great promise for cancer diagnosis, prognosis, and molecular characterization [31,32], most studies have relied on cfDNA extracted from plasma, where tumor-derived signals are often diluted by DNA from other tissues. In contrast, cfDNA from NAF offers a more localized and potentially enriched source of tumor-specific DNA. However, the use of NAF cfDNA in methylation studies remains underexplored. In this study, we demonstrated that high-quality sequencing libraries can be generated from nanogram-scale NAF cfDNA, achieving mapping rates exceeding 97% and genome coverage between 20× and 35×. This depth exceeds the 5–15× range recommended for reliable DMR detection in WGBS studies [33], supporting that our average coverage of 26.5× was sufficient for robust methylation calling.

To profile methylation across the genome, we employed cfBS, which offers several advantages over other high-throughput methylation technologies. Compared to MeDIP-seq [34,35], which uses antibody-based enrichment for methylated regions, cfBS provides base-pair resolution without bias toward heavily methylated sequences. Array-based methods such as the Illumina 450K and EPIC BeadChips offer high reproducibility and cost-efficiency, but they are limited to pre-selected CpG sites (~450,000 to 850,000 probes) and do not capture the full complexity of the methylome [36,37]. WGBS is the most comprehensive approach, capable of measuring the methylation status of every cytosine in the genome [38]. However, WGBS requires substantial DNA input and is cost-prohibitive for routine clinical use, particularly in liquid biopsy applications where DNA is limited [39]. In contrast, cfDNA is often enriched in coding and regulatory regions, possibly due to protection from nuclease degradation [13,40], making cfBS especially efficient for capturing functionally relevant methylation changes.

Our findings revealed 7471 DMRs between BrC and healthy samples, with a predominance of hypermethylation in cancer-associated gene regions, including several well-known tumor suppressors. Importantly, these methylation changes were enriched in gene promoters, suggesting a functional role in gene silencing and cancer progression, corroborating previous studies [41,42]. DNA methylation over the gene body is known to correlate positively with the level of gene transcription in the human genome [43,44]. To validate this, we used TCGA-BRCA data to confirm that a significant proportion of these DMGs were also differentially expressed, many with an inverse methylation-expression relationship, reinforcing the biological relevance of the observed methylation patterns.

A major finding of our study was the role of EMT during cancer progression. Using a novel EMT scoring method based on methylation profiles of curated EMT gene sets, we found that BrC cfDNA samples consistently exhibited a more mesenchymal phenotype than healthy controls. EMT-related pathways such as cell adhesion, PI3K-Akt, and Ras signaling were significantly enriched among the DMGs. Furthermore, the EMT scores correlated with tumor stage, supporting their potential prognostic value.

We also identified hypermethylation in several EMT-related genes with known roles in BrC. Promoter hypermethylation of *CDH1*, a hallmark of stable EMT [45], was observed through our findings and is consistent with its positive association with EMT in BrC cell lines [46]. This supports the notion that epigenetic silencing of *CDH1* contributes to reduced E-cadherin expression [47]. Similarly, *TRIM29* is often silenced in breast tumors due to aberrant gene hypermethylation and acts as a tumor suppressor through its ability to suppress EMT [48]. *WNT2*, a key ligand in the Wnt signaling pathway, also displayed promoter hypermethylation and has been suggested to play an important role in BrC tumorigenesis [49]. In addition, *COL5A2*, a gene related to extracellular matrix remodeling, exhibited hypermethylation within the coding region. Aberrant expression of *COL5A2* has been reported in BrC and is suggested to play a role in facilitating the invasiveness of BrC cells [50]. Furthermore, lncRNA-CASC15 and miR-129-5p emerged as potential epigenetic regulators. CASC15, an oncogenic factor in tumorigenesis of various cancers including BrC [51], is known to promote EMT by increasing N-cadherin and vimentin protein levels while decreasing that of E-cadherin via TWIST1 [52]. In our study, CASC15 exhibited promoter hypermethylation, which may represent an epigenetic mechanism contributing to its oncogenic activity. It has been reported that miR-129 is consistently downregulated in BrC samples, thereby regulating BrC cell proliferation and apoptosis [53]. Our observation of promoter hypermethylation of miR-129-5p provides a plausible mechanism for its downregulation, in line with reports demonstrating that downregulation of miR-129-5p through the Twist1-Snail feedback loop stimulates EMT [54]. These findings suggest that cfDNA methylation signatures in NAF not only reflect the presence of cancer but also capture molecular phenotypes indicative of disease aggressiveness.

Given the well-established inverse correlation between DNA methylation and gene expression [55], we were most interested in EMT-associated genes that demonstrate a negative correlation between these parameters. There was a significant overlap between DEGs identified in TCGA and DMGs in our cfBS data, with a substantial proportion of overlapping genes demonstrating concordant expression and methylation changes. Furthermore, consistent with TCGA data, our cfBS data highlighted differential methylation in key EMT-associated genes, supporting mechanisms that regulate the expression of genes previously linked to EMT. These findings suggest that cfDNA methylation patterns can be reliable indicators of tumor-specific epigenetic alterations. Functionally, *RABGAP1L* was shown to promote the invasive migration of BrC cells by facilitating the recycling of active *β*1 integrins [56]. As a known tumor suppressor, *AKAP12* inhibits the growth and metastasis of cancer cells [57]. Downregulation of *PGK1*, a key enzyme in aerobic glycolysis, was shown to suppress the invasion of BrC cells and reverse the EMT process [58]. *S100A14* has been identified as a modulator of HER2 signaling, with its overexpression significantly enhancing migration, invasion, and metastasis of BrC cells [59,60]. Similarly, overexpression of *TMPRSS4*, a serine protease expressed on the cell surface that contributes to the degradation of the extracellular matrix, has been suggested to promote tumor proliferation and aggressiveness in BrC [61]. These findings highlight the critical role of cfDNA differential methylation in regulating EMT-associated gene expression and driving BrC progression.

The major limitation of our study is the relatively small sample size, which limits the statistical power for identifying methylation differences and makes cross-validation unfeasible. To address this, we expanded our analysis by incorporating external validation using the TCGA-BRCA dataset, which includes both DNA methylation and gene expression data derived from a large cohort of primary BrC and normal tissue samples. This allowed us to validate key findings, such as DMGs and EMT signatures, in an independent dataset and to establish consistent correlations between methylation alterations and gene expression changes. Furthermore, we are currently planning a larger-scale study involving NAF cfBS profiling in a large patient cohort. This follow-up study will include a broader range of BrC subtypes and clinical stages, enabling more detailed biomarker discovery, subtype stratification, and assessment of diagnostic and prognostic performance.

Collectively, this study provides proof of concept that NAF cfDNA methylation profiling via WGBS is both feasible and informative, offering a powerful, noninvasive approach for BrC screening and molecular characterization. The ability to detect EMT activation from NAF cfDNA further adds functional insight that may guide risk stratification or therapeutic decisions. Moving forward, larger cohort studies are needed to validate these markers and assess their performance in early detection, particularly in high-risk or mammographically challenging populations. The integration of NAF-based liquid biopsy into clinical workflows could complement current screening tools and advance personalized, minimally invasive diagnostics for BrC.

## 4. Methods

### 4.1. Sample Collection and cfDNA Extraction

De-identified NAF samples from 7 individuals (3 with breast cancer and 4 healthy controls) were obtained from an established biobank. No new samples were collected for this study. The Texas A&M University Institutional Review Board (IRB) reviewed the project and determined that it does not constitute research involving human subjects. The NAF samples were gently drawn using a non-invasive breast pump, as described in [62], and subsequently stored in capillary tubes at a temperature of −80 °C. QIAamp Circulating Nucleic Acid Kit (Qiagen GmbH, Hilden, Germany) was used for the isolation of cfDNA. Briefly, the NAF samples were diluted in phosphate-buffered saline to reach the minimal required volume of 1 mL prior to extraction. The extraction procedure, comprising 4 steps (lyse, bind, wash, and elute), was carried out using QIAamp Mini columns (Qiagen GmbH, Hilden, Germany) on a vacuum manifold. The cfDNA was collected in a final elution volume of 20 µL. The concentration of the extracted cfDNA was quantified using the Qubit 1X dsDNA High Sensitivity Assay (Invitrogen, Thermo Fisher Scientific, Waltham, MA, USA).

### 4.2. cfDNA Bisulfite Sequencing

cfBS libraries were constructed using the Pico Methyl-Seq Library Kit (Zymo Research, Irvine, CA, USA). The process began with the bisulfite treatment of the input cfDNA, which simultaneously led to its random fragmentation. Post bisulfite conversion, the DNA underwent an initial amplification using random primers. This step was followed by the ligation of adaptors and a final amplification stage using Illumina TrueSeq indices (Illumina, San Diego, CA, USA). The amplified library size was assessed by Bioanalyzer High Sensitivity DNA Analysis (Agilent Technologies, Santa Clara, CA, USA) to validate the library quality. The library concentrations were measured using the Qubit 1X dsDNA High Sensitivity assay (Invitrogen, Thermo Fisher Scientific, Waltham, MA, USA). The samples were pooled at 8 nM and sequenced on Illumina HiSeq 2000 (paired-end 150, Illumina, San Diego, CA, USA).

### 4.3. Bioinformatics and Statistics

The sequenced reads were evaluated through FastQC (version 0.11.8). The mapping quality and sequencing coverage were assessed through samtools flagstat (version 1.9) and NGSEP CoverageStats (version 3.3.2), respectively. All qualified cfDNA sequenced reads were aligned to the Human Reference Genome Build GRCh38 (hg38) via bwa-meth (version 0.2.2). MethylDackel (version 0.5.2) was used to extract methylation levels from each sample.

The methylation level at each CpG site was calculated by the proportion of methylated reads relative to the total read count. The CpG islands were determined by clustering individual CpG sites using MethylC, an in-house mean shift-based machine learning program. This method iteratively shifts single CpG sites towards the highest density window with an initial size of 200 bp, and it locates the local maxima of mean methylation levels.

Fisher’s exact test was performed for differential methylation analysis. The mean difference in percentage methylation (%) was computed as the mean methylation ratio of cancer samples subtracted by the mean methylation ratio of normal samples. The DMRs between cancerous and normal samples were identified using stringent criteria: Fisher’s exact test *p*-value < 10^−8^, a total read count per CpG site twice exceeding the sample size (2n), and an absolute methylation difference ≥ 10%. The DMRs were annotated to genes according to their genomic locations. DMRs located within the region between the TSS and transcription end site (TES) were classified as being associated with the gene body. A promoter region was defined as being 5000 base pairs upstream of the TSS. Those sites outside of promoter regions or gene bodies were classified as intergenic.

A statistical test based on the geometric distribution was employed to determine whether the number of overlapping genes exceeded what was expected by chance. Single gene analysis was conducted to investigate the DMGs by assessing methylation levels at individual CpG sites within the corresponding DMR.

### 4.4. Gene Sets Analysis

PCA was conducted for dimensionality reduction and to identify the important PCs that effectively distinguished between the groups. For pathway analysis, we utilized ‘quickpath’ (version 0.0.0.9000), an R package developed by our lab [63,64]. This analysis uncovered biological processes significantly represented in DMGs, based on the Gene Ontology (GO) and the Kyoto Encyclopedia of Genes and Genomes (KEGG) database. A hypergeometric test, equivalent to a one-tailed version of Fisher’s exact test, was used to measure the significance of GO terms and biological pathways. The *p*-values were adjusted using the Benjamini–Hochberg method to control the false discovery rate (FDR) below 0.05, establishing our threshold for significance.

### 4.5. Computation of EMT Score

**Figure 7:**
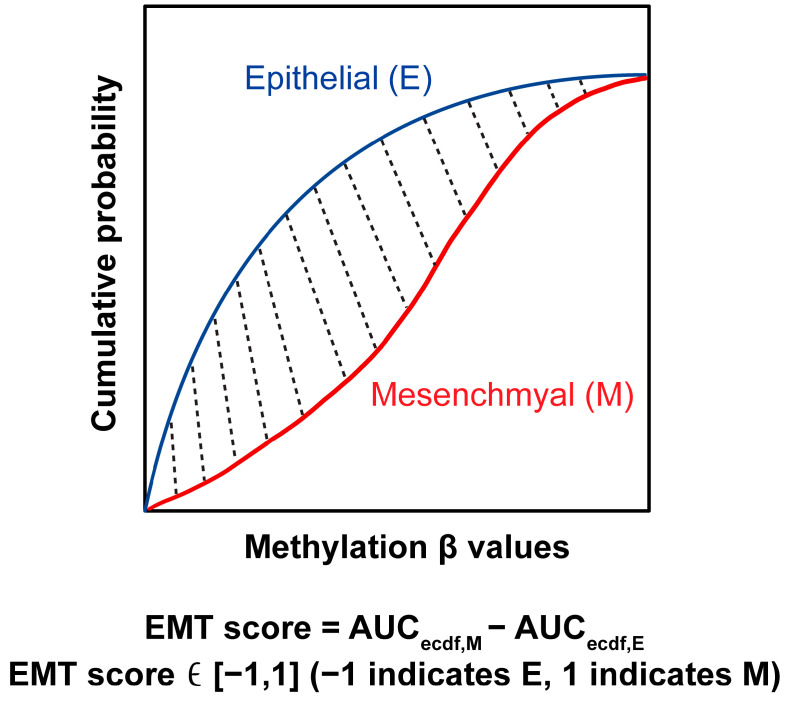
EMT scoring method. The blue curve indicates the empirical cumulative distribution function (ECDF) of epithelial gene signatures, while the red curve indicates the ECDF of mesenchymal signatures. The shaded area (EMT score) is derived by subtracting the AUC for epithelial genes from the AUC of mesenchymal genes.

We developed a method called EMT-Met for quantifying the EMT score in each sample based on the Kolmogorov–Smirnov method [65]. First, the methylation level for each sample was normalized to ensure uniformity in scoring. Secondly, we referenced the EMT genes from [66], which were pre-categorized into two groups: Epithelial (Epi; E) and Mesenchymal (Mes; M). There were 171 E genes and 52 M genes. The selected EMT genes are listed in Supplemental File S3: Table S2. Thirdly, for these biomarkers, we calculated the empirical cumulative distribution functions (ECDFs) based on their DNA methylation levels (β values). In Figure 7, the blue and red curves are representatives of Epi and Mes, respectively. Lastly, the AUC for both Epi and Mes curves was calculated. The EMT score (Figure 7 shaded area) was calculated by subtracting the AUC for Epi from the AUC for Mes, as follows:

EMT score = AUC_ecdf,M_ − AUC_ecdf,E_ (1)

In Equation (1), the score ranges from −1 to 1, with a positive score indicating an M phenotype, whereas a negative score is associated with the E phenotype. The EMT scoring was performed on MATLAB R2021b (MathWorks, Natick, MA, USA).

### 4.6. The Cancer Genome Atlas Breast Invasive Carcinoma Data Analysis

To validate the cfDNA findings, we analyzed the TCGA-BRCA dataset derived from tissue samples, focusing on EMT-associated genes identified in our study. RNA-sequencing data (raw read counts) and DNA methylation microarray data (methylation levels in β values) from the TCGA-BRCA dataset were obtained using TCGAbiolinks (version 2.34.0) in the R programming environment (version 4.4.1). Differential expression analysis was performed using the R limma package (version 3.62.1) by comparing 1111 tumor tissues with 114 adjacent normal tissues. DEGs were determined by the following criteria: absolute log2-based fold change ≥ 1 and a Bonferroni-adjusted *p*-value < 0.05. EMT score was calculated for each TCGA-BRCA sample using the EMT gene signatures previously identified from cfDNA methylation profiling. The β values, which represent the ratio of methylated probe intensity to the total probe intensity (both methylated and unmethylated), were used to calculate the EMT scores. To evaluate the discriminatory ability of the EMT score, we performed ROC curve analysis comparing tumor and normal samples using the pROC R package (version 1.18.5). For integrative analyses of gene expression and DNA methylation, 112 primary tumor and 84 normal solid tissue samples with both RNA-sequencing and methylation data were used. Pearson correlation analysis was performed between normalized gene expression values and methylation β values. The detailed clinical characteristics of the TCGA-BRCA cohort are provided in Supplemental File S4: Table S3.

## 5. Conclusions

This proof-of-concept study demonstrates the feasibility and potential of using cfDNA from NAF to detect BrC-associated DNA methylation signatures. We identified 7471 DMRs, the majority of which were enriched in gene regulatory regions, particularly promoters. This enrichment established a strong association between these epigenetic alterations and the gene expression changes observed in the TCGA-BRCA dataset. Our findings highlight the activation of the EMT program in BrC samples, supported by both methylation and transcriptomic data. By introducing a novel EMT scoring method based on cfDNA methylation patterns, we demonstrated that BrC samples exhibit a more mesenchymal phenotype, which correlates with increasing tumor stage. Together, these results underscore the clinical utility of NAF cfDNA methylation profiling as a noninvasive, localized, and informative approach for early BrC detection and molecular characterization. Future studies with larger cohorts are warranted to validate these findings and advance NAF-based liquid biopsy into routine clinical screening.

## Figures and Tables

**Table 1:** Sample information and the genome-mapping statistics of cfDNA bisulfite sequenced reads.

Sample	Cancer Status	Race	Age	No. of Total Reads	No. of Total Mapped Reads	% Mapping	Sequence Depth	Percent Coverage
1	Benign	White	46	129,617,861	127,235,741	98.2%	22.3×	53.4%
2	Benign	White	37	116,237,181	112,740,144	97.0%	27.4×	38.4%
3	Benign	White	54	157,025,790	154,506,008	98.4%	33.9×	42.5%
4	Benign	White	38	104,337,726	102,763,750	98.5%	21.5×	44.6%
5	Cancer	White	51	136,874,162	135,539,287	99.0%	23.6×	53.8%
6	Cancer	White	50	203,458,690	200,828,216	98.7%	31.9×	58.8%
7	Cancer	White	40	107,615,356	106,564,363	99.0%	24.7×	40.3%

## Data Availability

The datasets used in this study are available through the Gene Expression Omnibus (GEO) database with GEO accession number GSE238014. The R package ‘quickpath’ is available on Github: https://github.com/jiangyuan2li/quickpath (accessed on 6 September 2025). The code for MethylC is available on Github: https://github.com/minsunsjeon/MethylC (accessed on 6 September 2025). The code for EMT-Met is available on Github: https://github.com/minsunsjeon/EMT-Met (accessed on 6 September 2025).

## Supplementary Materials

The following supporting information can be downloaded at: https://www.mdpi.com/article/10.3390/ijms26178723/s1.

## Declarations

Opinions expressed by the authors are their own, and this material should not be interpreted as representing the official viewpoint of the US Department of Health and Human Services, the National Institutes of Health, the National Cancer Institute, or the Division of Cancer Prevention.

## Abbreviations

The following abbreviations are used in this manuscript:

AUC	Area Under the Curve
BrC	Breast Cancer
cfBS	cfDNA Bisulfite Sequencing
cfDNA	Cell-free DNA
DEG	Differentially Expressed Gene
DMG	Differentially Methylated Gene
DMR	Differentially Methylated Region
ECDF	Empirical Cumulative Distribution Function
EMT	Epithelial–Mesenchymal Transition
Epi	Epithelial
FDR	False Discovery Rate
GO	Gene Ontology
KEGG	Kyoto Encyclopedia of Genes and Genomes
Mes	Mesenchymal
MSP	Methylation-Specific PCR
NAF	Nipple Aspirate Fluid
PC	Principal Component
PCA	Principal Component Analysis
ROC	Receiver Operating Characteristic
TCGA-BRCA	The Cancer Genome Atlas Breast Invasive Carcinoma
TES	Transcription End Site
TSS	Transcription Start Site
WGBS	Whole-Genome Bisulfite Sequencing
