# Supplementary figures and images for: Cell-Free DNA Bisulfite Sequencing Reveals Epithelial–Mesenchymal Transition Signatures for Breast Cancer
Source: Int J Mol Sci. 2025 Sep 7;26(17):8723. doi: 10.3390/ijms26178723 (PMC12429146; doi:10.3390/ijms26178723)

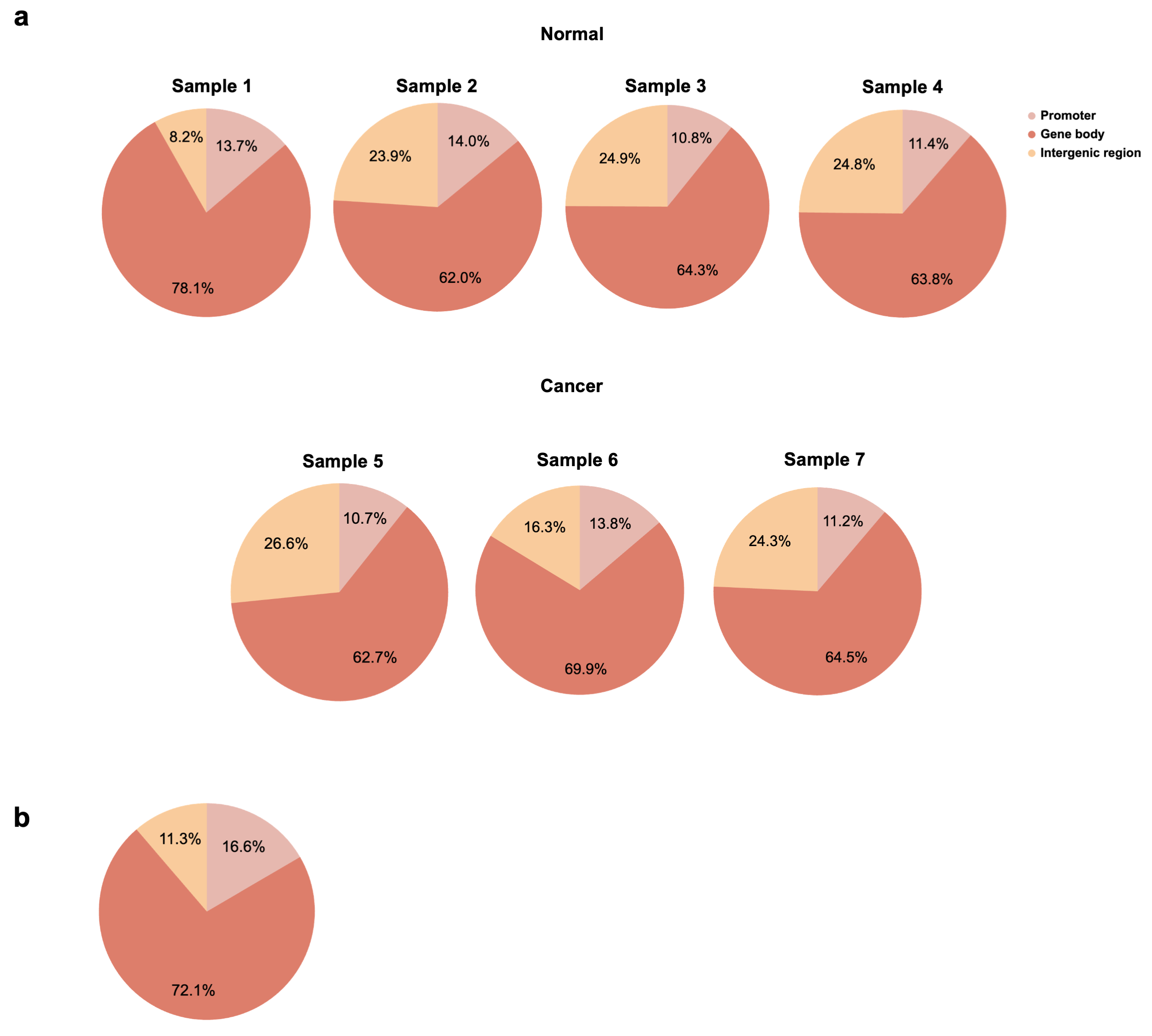

Supplement: Supplementary file 1 [file ijms-26-08723-s001.zip › Figure S1.tif]
